# Adenylate kinase-independent thiamine triphosphate accumulation under severe energy stress in Escherichia coli

**DOI:** 10.1186/1471-2180-8-16

**Published:** 2008-01-23

**Authors:** Tiziana Gigliobianco, Bernard Lakaye, Alexander F Makarchikov, Pierre Wins, Lucien Bettendorff

**Affiliations:** 1 Center for Cellular and Molecular Neurobiology, University of Liège, Liège, Belgium; 2 Chemical Department, Grodno State Agricultural University, Grodno, Belarus

## Abstract

**Background:**

Thiamine triphosphate (ThTP) exists in most organisms and might play a role in cellular stress responses. In E. coli, ThTP is accumulated in response to amino acid starvation but the mechanism of its synthesis is still a matter of controversy. It has been suggested that ThTP is synthesized by an ATP-dependent specific thiamine diphosphate kinase. However, it is also known that vertebrate adenylate kinase 1 catalyzes ThTP synthesis at a very low rate and it has been postulated that this enzyme is responsible for ThTP synthesis in vivo.

**Results:**

Here we show that bacterial, as vertebrate adenylate kinases are able to catalyze ThTP synthesis, but at a rate more than 10^6^-fold lower than ATP synthesis. This activity is too low to explain the high rate of ThTP accumulation observed in E. coli during amino acid starvation. Moreover, bacteria from the heat-sensitive CV2 strain accumulate high amounts of ThTP (>50% of total thiamine) at 37°C despite complete inactivation of adenylate kinase and a subsequent drop in cellular ATP.

**Conclusion:**

These results clearly demonstrate that adenylate kinase is not responsible for ThTP synthesis in vivo. Furthermore, they show that E. coli accumulate large amounts of ThTP under severe energy stress when ATP levels are very low, an observation not in favor of an ATP-dependent mechanisms for ThTP synthesis.

## Background

Thiamine (vitamin B1) is an essential compound for all known life forms. The well-known cofactor thiamine diphosphate (ThDP) [1] is the major form of thiamine in most cell types. Thiamine monophosphate (ThMP) and free thiamine, which have no known physiological function, account for only a few percent of the total thiamine content. Thiamine triphosphate (ThTP) is generally a minor component but it has been found in most organisms, from prokaryotes to mammals [2]. In vertebrates, ThTP has been found to activate a large conductance anion channel [3] and to phosphorylate certain proteins [4], suggesting that it may be involved in a new cellular signaling pathway.

In animal tissues, cellular concentrations of ThTP generally remain relatively constant and low (0.1 to 1 μM). In contrast, in the enterobacterium E. coli, ThTP content strongly depends on environmental conditions. ThTP is nearly undetectable in rich LB medium, but in minimal medium devoid of amino acids, the addition of a carbon source such as glucose or pyruvate induces a rapid accumulation of ThTP and its intracellular concentration may transiently exceed 10 μM [5]. Overexpression in E. coli of a specific soluble mammalian thiamine triphosphatase (ThTPase), that we previously characterized [6-8], prevented ThTP accumulation and induced the appearance of an intermediate plateau in bacterial growth [5]. This suggested that ThTP may be required for the rapid adaptation of bacteria to amino acid starvation. On the other hand, when the bacteria were incubated in minimal medium devoid of any carbon source, we noticed the appearance of a new compound that was identified as adenosine thiamine triphosphate (AThTP) [9]. Interestingly, ThTP and AThTP never accumulate simultaneously in high amounts, suggesting that the two compounds may act as specific alarmones, responding to different conditions of cellular stress.

While AThTP is synthesized according to the recently established reaction ThDP + ADP (ATP) ⇔ AThTP + P_i _(PP_i_) [10], the enzymatic mechanism of ThTP synthesis remains unclear. It has been shown that vertebrate adenylate kinase 1 (AK1, myokinase, EC 2.7.4.3) catalyzes the synthesis of ThTP at a low rate according to the reaction ThDP + ADP ⇔ ThTP + AMP [11]. Although, the in vivo synthesis of ThTP by AK1 was shown to occur in chicken skeletal muscle [12], we have found that AK1 knockout mice have normal ThTP levels (even in skeletal muscle). This suggests that ThTP synthesis by AK1 is not of physiological relevance in mammals [13], which does not rule out that other mammalian AK isoforms [14] may be responsible for ThTP synthesis. Here we show that two bacterial AKs are able to catalyze ThTP synthesis at a low rate but our data strongly suggest that this enzyme is not responsible for the in vivo accumulation of ThTP in E. coli in response to amino acid starvation.

## Results and Discussion

We have previously reported [5] that when E. coli cells are transferred to a minimal medium containing glucose, they accumulate ThTP at a high rate (about 100 pmol per mg protein in 10 min). As AK is a possible candidate for catalyzing ThTP synthesis, it is important to determine whether bacterial AKs are able to catalyze this reaction and, if they do, to know whether the specific activity of, in particular, E. coli AK is sufficient to account for the relatively high rate of ThTP production measured in vivo in this organism.

We first tested the ability of commercially available AK from B. stearothermophilus to synthesize ThTP from ThDP and ADP. The enzyme was indeed able to catalyze this reaction at a rate of 0.2 pmol ThTP formed per min per mg protein under our assay conditions. For the physiological reaction 2 ADP ⇔ ATP + AMP, we found 1.8 μmol min^-1 ^mg^-1^. Thus, ThTP synthesis by this enzyme is 10^7 ^times slower than ATP synthesis (Table 1).

**Table 1:** Comparison of ThTP- and ATP-synthesizing activity of adenylate kinases from various sources.

Sources	ThTP synthesis (pmol min^-1 ^mg^-1^)	ATP synthesis (pmol min^-1 ^mg^-1^)	Ratio ATP/ThTP
AK B. stearothermophilus^a^	0.2 (pH 6.5)	1.8 × 10^6^	9 × 10^6^
AK E. coli^a^	35	137 × 10^6^	3.9 × 10^6^
AK1 pig skeletal muscle^b^	570	1055 × 10^6^	1.8 × 10^6^
AK1 chicken skeletal muscle^c^	60	155 × 10^6^	2.6 × 10^6^
Ibid.	265 (pH 10)	155 × 10^6^	0.6 × 10^6^

ThTP synthesis was measured at pH 7.5, except when indicated, as described in the Methods section. The assays for ATP synthesis were done at pH 7.5. ^a^, this study; ^b^, [11]; ^c^, [16]

**Figure 1:**
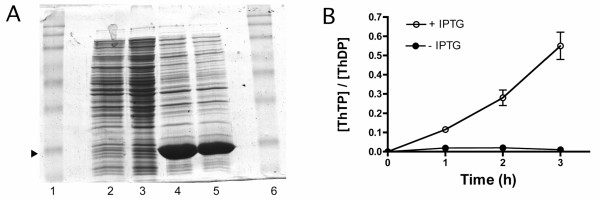
**Overexpression of E. coli AK induces cellular ThTP accumulation**. Overexpression of E. coli AK induces cellular ThTP accumulation. Strain BL21 λDE3 bacteria carrying pET-21a(+) with the E. coli AK cDNA were grown ovenight in LB medium in the presence of ampicillin (1 mg/ml). The cultures were diluted until A_600 _= 0.6 – 0.8 and the bacteria were grown in the absence or presence of IPTG (1 mM) for 3 h (37°C, 250 rpm). The bacteria were sonicated, centrifuged and the supernatant was used for electrophoresis (A) and ThTP estimation (B). (A) The presence of a band corresponding to AK was checked on a 12% SDS-PAGE followed by Coomassie blue staining (a, lanes 2, 3 without IPTG and lanes 4, 5 with IPTG). Lanes 1 and 6 contain the molecular weight markers (Prestained Protein Ladder, Invitrogen). Overexpessed protein migrated at M_r _25 000 (arrowhead) for a theoretical molecular mass of 23559 based on amino acid composition [15]. (B) Effect of E. coli AK overexpression on the intracellular [ThTP]/[ThDP] ratio. Aliquots were taken at various times for the determination of thiamine derivatives. The results are expressed as mean ± SD for 3 experiments. At the same time ATP synthesizing activity of AK increased by a factor 1000 in cell-free extracts (not shown).

E. coli has only one AK isoform [15]. We overexpressed this protein in E. coli BL21 λDE3 under the control of the lac operon. As shown in Fig. 1A, the transformed bacteria produced high amounts of AK after induction by IPTG. At the same time, the ATP-synthesizing activity of E. coli AK was increased about 1000-fold (from 67 nmol min^-1 ^mg^-1 ^to over 50 μmol min^-1 ^mg^-1^) in cell-free extracts. We also observed that these bacteria began to accumulate ThTP shortly after addition of IPTG (Fig. 1B), suggesting that ThTP synthesis is a direct consequence of AK overexpression. After 3 hours, the [ThTP]/[ThDP] ratio reached a relatively high value around 0.55.

However, it is important to emphasize that in contrast to the normal BL21 strain, which accumulates ThTP only in the presence of glucose in amino acid-depleted medium, E. coli overexpressing AK accumulated ThTP in rich LB medium and glucose was not required. Furthermore, IPTG did not induce ThTP synthesis in control bacteria carrying an empty plasmid (not shown). These results suggest that E. coli AK constitutively synthesizes ThTP and that there is no physiological control of this reaction. The present results are very similar to those of Shioda et al. [16] who overexpressed chicken AK1 in E. coli and also observed an important accumulation of ThTP after induction by IPTG in rich LB medium. We measured the ThTP-synthesizing activity of the overexpressed bacterial AK in vitro, in the supernatant obtained after sonication and centrifugation of the bacteria. Under our assay conditions the rate was 35 pmol min^-1 ^mg^-1^, while the rate of ATP synthesis was 137 μmol min^-1 ^mg^-1^. Thus, ThTP synthesis catalyzed by E. coli AK is over 6 orders of magnitude slower than ATP synthesis (Table 1). A similar ratio between ThTP and ATP synthesis was also previously reported for porcine [11] and chicken AK1 [16] and for B. stearothermophilus AK (this study).

We conclude that the three types of AK investigated (AK1 from vertebrates, AK from B. stearothermophilus and AK from E. coli) are able to catalyze ThTP synthesis from ThDP and ADP, but the reaction is over 10^6 ^times slower than ATP synthesis. It is thus possible that the catalysis of ThTP synthesis is a general property of AKs, but this reaction is not likely to be of physiological importance.

It is interesting to compare the rate of ThTP synthesis by AKs in vitro with the maximum rate observed in vivo. The latter is about 10 pmol min^-1 ^mg^-1 ^[5] for normal BL21 bacteria in minimal medium containing 10 mM glucose. In cell-free extracts from BL21 bacteria, we found that the specific activity for AK-catalyzed ATP synthesis was 67 nmol min^-1 ^mg^-1^. Assuming that the rate of ThTP synthesis is 10^6^ times lower, it would be about 0.05 – 0.10 pmol min^-1 ^mg^-1^, two orders of magnitude lower than the accumulation measured in vivo. But after induction by IPTG, AK expression is increased about 1000-fold (Fig. 1A), largely enough to account for the observed ThTP accumulation (Fig 1B). However, the ThTP-forming activity of bacterial AK appears to be constitutive and to escape physiological control. Nevertheless, it could still be argued that bacterial AK is responsible for ThTP synthesis in vivo if one assumes that the enzyme can be activated (≥100-fold) by some unknown factor(s).

**Figure 2:**
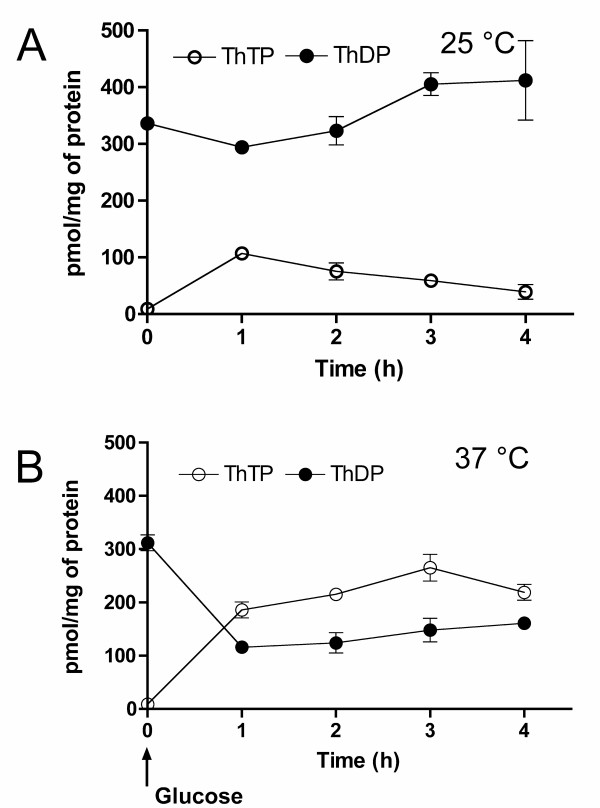
**Effect of AK inactivation in the heat-sensitive E. coli strain CV2 on the intracellular ThTP and ThDP concentrations**. Effect of AK inactivation in the heat-sensitive E. coli strain CV2 on the intracellular ThTP and ThDP concentrations. The bacteria were grown at 25°C in LB medium, suspended in minimal M9 medium and preincubated for four hours either at 25°C (A) or at 37°C (B). After 60 min at 37°C, no significant AK activity was detectable, while it remained high at 25°C (not shown). Then glucose was added at 25 and 37°C (zero time) and aliquots were taken after various time intervals for the determination of thiamine derivatives. The results are expressed as mean ± SD (n = 3).

The following experiment strongly suggests that a different, AK-independent mechanism is responsible for ThTP synthesis in E. coli. We used the CV2 strain [15,17] containing a heat-sensitive AK. CV2 E. coli grow normally at the permissive temperature (25 – 30°C) but shifting them to 37°C leads to a rapid inactivation of AK and the subsequent drop in energy charge (from 0.9 to 0.2, [18]), causing a progressive decrease in the number of viable cells. As shown in Fig. 2A, CV2 bacteria grown at 25°C respond normally by producing ThTP when they are transferred to minimal medium containing glucose. However, high ThTP levels were maintained for at least 2 hours, while in BL21 bacteria incubated at 37°C, ThTP accumulation was transient [5]. When the CV2 bacteria were shifted to 37°C, they lost all detectable AK activity after less than 2 hours as previously reported [18]. However, addition of glucose under these conditions led to an unexpectedly high production of ThTP. As shown in Fig. 2B, the cellular ThTP content after 2 hours even exceeded ThDP content while the total amount of phosphorylated thiamine, i.e. [ThDP] + [ThTP], remained roughly constant.

These results show that high amounts of ThTP (60% of total thiamine) can be synthesized from ThDP in the absence of AK activity. The phosphate donor is therefore unlikely to be ADP. Another obvious candidate is ATP. However, when we incubate CV2 bacteria at 37°C we find that, even in the presence of glucose, the cellular ATP concentration is less than 10% of the one found in normal BL21 bacteria or CV2 bacteria at 25°C (data not shown) as previously observed [18]. This is not in favor of the hypothesis that ATP is the phosphate donor except if the putative ThDP kinase catalyzing this reaction has a very high affinity for ATP.

Actually, there is so far no evidence that E. coli contains a ThDP kinase. In cell-free bacterial extracts, we attempted to measure ThTP formation from ThDP and ATP under various conditions but we were unable to detect any net synthesis of ThTP. It thus appears that the phosphate donor for ThDP phosphorylation may be neither ADP nor ATP, at least in E. coli. In eukaryotic organisms, it has long been thought that ThTP is synthesized by a soluble ThDP kinase: this enzyme was supposed to exist in mammals [19-22] and in yeast [23,24]. The enzyme was obtained in pure form from yeast [23] but, like other preparations of ThDP kinase, it had a very low specific activity (k_cat _about 1 min^-1^). Moreover, it is not certain that the reaction product was authentic ThTP. Indeed, it could well be that the compound synthesized was in fact AThTP which can be synthesized from ThDP and ATP or ADP by a soluble enzyme complex [10]. Thus, there is no conclusive evidence that ThTP can be synthesized from ThDP + ATP, either in animals or in microorganisms. Also, it is probable that in skeletal muscle where AK1 activity is very high, the latter may contribute to a significant synthesis of cytosolic ThTP, especially in those species where soluble ThTPase activity is absent such as in electric organ [25,26], chicken [12] and pig skeletal muscle [27]. Indeed, electric organs and bird tissues contain no soluble ThTPase and pig tissues express a catalytically inefficient ThTPase [28].

## Conclusion

In conclusion, the present results show that, in the heat-sensitive CV2 strain as in normal E. coli, ThTP accumulation occurs through an adenylate kinase-independent mechanism. The bacteria produce ThTP when they are transferred to minimal medium devoid of amino acids but containing glucose. The requirement for glucose does not appear to be related to its ability to generate ATP. Indeed, we find the highest accumulation of ThTP in CV2 cells at 37°C when the energy charge is very low. It is remarkable that under such stressful conditions, the cells still devote a large part of their ThDP (an indispensable cofactor for oxidative metabolism) and a significant amount of free energy to produce ThTP. At present, we cannot exclude that ThTP and possibly AThTP are inactive storage forms of ThDP. This hypothesis is however not very plausible as ThTP and AThTP accumulate under different and often opposing metabolic conditions. This would imply that ThDP could be stored under a different form dependent on the kind of stress involved. Therefore, it is more appealing to imagine that both compounds are some kind of alarmones or signalling molecules produced in response to different conditions of cellular stress.

## Methods

### Materials

B. stearothermophilus AK was from Sigma-Aldrich (St-Louis, MO, USA). The heat-sensitive E. coli strain CV2 (CGSC strain # 4682) [17] was obtained from the E. coli Genetic Resource Center (Yale University, New Haven, CT, U.S.A.) through N. Whitehead. It was grown at 25°C in LB medium (250 rpm).

### Growth and processing of bacteria

The bacteria (E. coli BL21 strain) were grown overnight (37°C, 250 rpm) in 50–100 ml LB medium (tryptone, 10 g/l; yeast extract, 5 g/l; NaCl, 10 g/l at pH 7.0). Then the bacteria were centrifuged (5 min; 5000 × g) and suspended in the initial volume of fresh LB medium or in M9 minimal medium (Na_2_HPO_4_, 6 g/l; KH_2_PO_4_, 3 g/l; NaCl, 0.5 g/l; NH_4_Cl, 1 g/l; CaCl_2_, 3 mg/l; MgSO_4_, 1 mM, pH 7.0) either in the presence or the absence of 10 mM glucose at 37°C with shaking (250 rpm). After incubation, the bacteria were sedimented as above, the pellet was suspended in 12% trichloroacetic acid, the precipitated proteins were spun down (15 min, 15 000 × g) and the pellet was dissolved in 0.8 N NaOH for protein determination by the method of Peterson [29]. The supernatant was treated with diethyl ether and analyzed by HPLC for thiamine compounds [30]. ATP was determined using the ATP Bioluminescent Assay Kit from Sigma-Aldrich.

### Cloning and overexpression of E. coli adenylate kinase

Genomic DNA was isolated from E. coli (BL21) and the coding sequence for adenylate kinase was amplified using Taq DNA polymerase and 40 cycles of denaturation (95°C, 30 s), annealing (58°C, 30 s) and elongation (72°C, 60 s) using forward (5'-CACATATGCGTATCATTCTGCTTGGCGCT-3') and reverse (5'-CAAAGCTTAGCCGATTTTTTCCAGATCAGCG-3') primers. The PCR fragment was inserted into pGEM-T (Promega Corporation, Madison, WI, U.S.A.) by TA cloning. After sequencing, the AK coding sequence was recovered and ligated into the NdeI/HindIII sited of pET-21a(+) (Novagen, Madison, WI, U.S.A). The strain E. coli BL21 λDE3 was used for overexpression of E. coli adenylate kinase.

### Determination of adenylate kinase activity

The culture medium containing the bacteria (1 ml) was centrifuged (5000 × g, 15 min, 4°C) and the pellet was suspended in 500 μl Hepes-Na buffer (50 mM, pH 7.5) containing 1 mM EDTA. The samples were sonicated 3 × 1 min on ice (100 kHz) and centrifuged (5000 × g, 10 min, 4°C). The supernatant was used as enzyme preparation. The incubation medium contained 50 mM Tris/HCl buffer (pH 7.5), 5 mM MgCl_2_, 5 mM ADP and the enzyme preparation at an appropriate dilution in a total volume of 100 μl. After 5 min at 37°C, the reaction was stopped by addition of 100 μl trichloroacetic acid (20%). After extraction with diethyl ether, ATP was determined by bioluminescence. For the determination of the ThTP-synthesizing activity of adenylate kinase, the substrates were ADP (1 mM) and ThDP (0.1 mM) in Tris/HCl buffer (pH 7.5) for E. coli AK and Tris-maleate buffer (pH 6.5) for B. stearothermophilus AK. The samples were incubated up to 24 hours and the ThTP synthesized was determined by HPLC [30].

## Authors' contributions

TG did most of the experimental work described in the study. BL oversaw the cloning of E. coli AK and participated in the design of the study. AFM participated in the initial experimental work with bacterial adenylate kinases. PW participated in the design of the study and revised the manuscript. LB was the project leader, coordinated the study, participated in its design and wrote the final manuscript. All authors read and approved the final manuscript.
